# Chronic Stress and the Development of Early Atherosclerosis: Moderating Effect of Endothelial Dysfunction and Impaired Arterial Elasticity

**DOI:** 10.3390/ijerph6122934

**Published:** 2009-11-27

**Authors:** Nadja Chumaeva, Mirka Hintsanen, Niklas Ravaja, Markus Juonala, Olli T. Raitakari, Liisa Keltikangas-Järvinen

**Affiliations:** 1 Department of Psychology, University of Helsinki, P.O. Box 9, 00014 Helsinki, Finland; E-Mails: nadezda.chumaeva@helsinki.fi (N.C.); mirka.hintsanen@helsinki.fi (M.H.); 2 Division of Medical Problems of Cell Biology, Institute of Cell Biophysics, Institutional Street 3, Pushchino, Moscow region 142290, Russia; 3 Center for Knowledge and Innovation Research, Helsinki School of Economics, P.O. Box 1210, 00101 Helsinki, Finland; E-Mail: niklas.ravaja@hse.fi; 4 Research Centre of Applied and Preventive Cardiovascular Medicine, University of Turku, Kiinanmyllynkatu 10, 20520 Turku, Finland; E-Mails: markus.juonala@utu.fi; 5 Department of Clinical Physiology, University of Turku, P.O. Box 52, 20521 Turku, Finland; E-Mail: olli.raitakari@utu.fi; 6 Department of Medicine, University of Turku and Turku University Hospital, P.O. Box 52, 20521 Turku, Finland

**Keywords:** chronic stress, vital exhaustion, intima-media thickness, flow-mediated dilatation, carotid artery compliance, atherosclerosis

## Abstract

This study aims to explore the interactive effect of vital exhaustion (VE) and endothelial dysfunction on preclinical atherosclerosis, assessed by carotid intima-media thickness (IMT). Furthermore, interaction between VE and carotid elasticity is examined. Participants were 1,596 young healthy adults from the Cardiovascular Risk in Young Finns study. Endothelial dysfunction was measured by brachial flow-mediated dilatation (FMD), and carotid elasticity by carotid artery compliance (CAC). Significant interactions between FMD and VE, and between CAC and VE, for IMT were found in participants with the very lowest FMD and CAC. Thus, VE may be harmful if the endothelium is not working properly.

## 1. Introduction

Atherosclerosis is a chronic progressive disease with a long preclinical phase. Risk factors such as age, smoking, blood pressure, hypercholesterolemia, hyperglycemia, diabetes, obesity, hypertension, infection/inflammation, and genetic factors are associated with the development of atherosclerosis, and are known to impair endothelial function [1], a key factor in the early atherogenic process [2]. Recent studies have shown that psychosocial factors may accelerate the development of carotid atherosclerosis [3].

Impaired endothelial function triggers the first step of atherosclerosis progression and manifests long before the establishment of atherosclerotic disease [2]. Non-invasive ultrasound measurement of brachial artery diameter changes due to reactive hyperemia-induced endothelial nitric oxide (NO) release is used as a method for evaluating endothelial function [4]. The change in diameter before and during hyperemia has been taken as an index of endothelial NO production and called flow-mediated dilatation (FMD) [4]. Reduced FMD has been shown to be related to the frequency and degree of coronary atherosclerosis [5]. Thus, FMD reflects the vascular endothelium status and may be used as a functional marker of atherosclerotic risk and as one of the endothelial health markers [6].

Carotid artery compliance (CAC) is widely used as an index of arterial elasticity [7]. Small artery compliance has been considered as an early marker for cardiovascular diseases [7], and it may be an additional indicator of early atherosclerosis [7,8].

Carotid intima-media thickness (IMT) has been shown to correlate to the severity of generalized atherosclerosis [9]. Increased carotid IMT is associated with coronary atherosclerosis [10] and with the standard cardiovascular risk factors [1].

Thus, carotid IMT, arterial compliance, and brachial FMD are important indexes of preclinical cardiovascular disease [7], and may serve as the markers of vascular health [11]. Individuals having impaired FMD and a reduced arterial compliance may be considered to have decreased vascular health and they are at an increased coronary risk [1,12]. The vascular health status may be suggested to influence the association between risk factors and atherosclerosis.

Chronic stress has been shown to be a risk factor for atherosclerosis development [13], and it has been found to contribute to endothelial dysfunction [14]. Vital exhaustion (VE) is determined as a psychological state which can be characterized by heightened irritability, unusual tiredness, a loss of physical and mental energy, and demoralized feelings [15]. It has been considered as an indicator of chronic mental stress [16] and reflects a decreased stress coping [15,16]. A number of studies have demonstrated VE after myocardial infarction [17], and after stroke [16]. Moreover, exhaustion has considered being of importance for rehabilitation [18], and treating exhaustion has been suggested as a strategy to improve the cardiovascular health [19]. Our recent study has shown that VE may contribute to an increased risk of early atherosclerosis in young healthy adults [20].

The combined effects of VE and endothelial function on atherosclerosis have not been investigated. The current study was designed to examine this issue. That is, we investigated the interactive effect of VE and endothelial dysfunction, measured by FMD, on preclinical atherosclerosis, assessed by carotid IMT, in young healthy adults aged 24–39 years. Likewise, we examined the interactive effect of VE and CAC on carotid IMT. Based on previous findings that VE is associated with heart diseases [15–18], and endothelial dysfunction (low FMD) or reduced arterial elasticity (reduced CAC level) are associated with cardiovascular risk [1], we hypothesized (1) that a high level of VE in combination with impaired endothelial function (low FMD) or lower arterial elasticity (reduced CAC level) may be related to preclinical atherosclerosis. Furthermore, we hypothesized (2) that these interactions would be most strongly present at the very lowest levels of FMD and CAC and (3) that traditional cardiovascular risk factors would mediate the association.

To evaluate whether the cardiovascular risk factors may mediate the interactive effect of VE and FMD on carotid IMT or may contribute to the interactive effect of VE and CAC on IMT, we took into account the effects of cardiovascular risk factors associated with ultrasound variables in Cardiovascular Risk in Young Finns (CRYF) study [21–23], and some other cardiovascular risk factors found important. Thus risk factors, such as serum LDL-cholesterol levels, serum HDL-cholesterol levels, triglyceride levels, serum insulin level, systolic and diastolic blood pressure, were included in the current study.

## 2. Methods

### 2.1. Subjects

The participants are from the sample of ongoing prospective epidemiological Cardiovascular Risk in Young Finns (CRYF) study [24]. The CRYF study was started in 1980 to investigate the development of risk factors for coronary heart disease in children, adolescents and young adults from different parts of Finland. Originally 3,596 healthy Finnish children, adolescents, and young adults aged 3–18 years participated in the first cross-sectional study in 1980. The design of the CRYF study has recently been described in detail [24]. In the original CRYF sample, carotid IMT, CAC, and brachial FMD were measured in 2001. Complete data on carotid and brachial artery ultrasound measurements were available for 2,109 subjects aged 24 to 39 years (in 2001). Valid VE questionnaires were obtained from 2,080 participants (in 2001). The final sample comprised 1,596 subjects. The study followed the guiding principles of the Helsinki Declaration and was approved by the local ethics committees. All subjects gave their written, informed consent.

### 2.2. Vital Exhaustion

Vital exhaustion was assessed with the Maastricht Questionnaire, a 21-item checklist of signs and symptoms of exhaustion [25]. Maastricht Questionnaire has been designed for self-application and specially developed to assess the feelings of vital exhaustion [25]. Cronbach’s alpha was 0.92, indicating good reliability. Each of the items was rated on a three-point scale, ranging from 0 to 2. The mean score of all items was used to index the level of VE. The questionnaire was sent to the participants to be completed at home.

### 2.3. Ultrasound Imaging

Ultrasound studies of the carotid and brachial arteries were performed using Sequoia 512 ultrasound mainframes (Acuson, Mountain View, CA, USA) with 13.0 MHz linear array transducer, as previously described [1,21–23]. To assess intra-individual reproducibility of ultrasound measurements, 57 subjects were re-examined three months after the initial visit (2.5% random sample) [1].

### 2.4. Carotid Intima-Media Thickness, IMT

Carotid IMT was measured on the posterior (far) wall of the left carotid artery. At least four measurements were taken approximately 10 mm proximal to the bifurcation to derive mean carotid IMT. The between-visit coefficient of variation (CV) of IMT measurements was 6.4% and the intra-observer CV was 3.4% [1].

### 2.5. Brachial Flow-Mediated Dilatation, FMD

To assess brachial FMD, the left brachial artery diameter was measured both at rest and during reactive hyperemia. Increased flow was induced by inflation of a pneumatic tourniquet placed around the forearm to a pressure of 250 mm Hg for 4.5 min, followed by a release [22]. Arterial diameter was measured at end-diastole at a fixed distance from an anatomic marker at rest and 40, 60 and 80 s after cuff release. The vessel diameter in scans after reactive hyperemia was expressed as the percentage relative to resting scan. The average of three measurements at each time point was used to derive the maximum FMD (the greatest value between 40 to 80 s). All ultrasound scans were analyzed by a single reader blinded to the subject’s details. The between-visit coefficient of variation for brachial diameter was 3.2% and for FMD 26.0% [22]. Intra-observer CV was 1.2% for brachial diameter, and 15.3% for FMD [1].

### 2.6. Carotid Artery Compliance, CAC

Several moving image clips of the beginning of the carotid bifurcation and the common carotid artery with a duration of 5 s were acquired and stored in digital format for subsequent offline analysis. The best quality cardiac cycle was selected from the clip images. The carotid diameter was measured at least twice (spatial measurements) in end-diastole and end-systole, respectively. The mean of the measurements was used as the end-diastolic or end-systolic diameter. Blood pressure was measured during the ultrasound study with an automated sphygmomanometer (Omron M4, Omron Matsusaka Co., Ltd, Japan). Ultrasound and concomitant brachial blood pressure measurements were used to calculate carotid compliance (CAC = [(Ds – Dd)/Dd]/(Ps – Pd), where Dd is diastolic diameter, Ds is systolic diameter, Ps is systolic blood pressure, and Pd is diastolic blood pressure). The between-visit CV was 2.7% for diastolic diameter and 16.3% for CAC [23]. Intra-observer CV was 1.4% for diastolic diameter, and 13.6% for CAC [1].

### 2.7. Clinical Characteristics and Cardiovascular Risk Factors

We took into account the effects of serum LDL-cholesterol levels, serum HDL-cholesterol levels, triglyceride levels, serum insulin level, systolic and diastolic blood pressure. For the determination of serum lipoprotein levels, venous blood samples were drawn after an overnight fast. All determinations were done using standard methods [26]. All measurements of lipid levels were performed in duplicate in the same laboratory. Serum insulin was measured by microparticle enzyme immunoassay kit (Abbott Laboratories, Diagnostic Division, Dainabot, Japan). Blood pressure was measured with a random-zero sphygmomanometer. Average of three measurements was used in the analysis. Blood pressure was included into analyses because it had previously been shown to predict IMT among young adults from 33 to 39 years [21].

### 2.8. Statistical Analyses

The interactions between FMD and VE, and between CAC and VE, in predicting carotid IMT were tested using linear regression techniques (SPSS Version 16.0). In the first model, the main effects of age, sex, VE, FMD, and baseline brachial diameter, as well as the interaction between FMD and VE, were included in the regression analyses when examining the interaction between FMD and VE in predicting IMT. In the second model, the main effects of the risk factors (serum LDL-cholesterol levels, serum HDL-cholesterol levels, triglyceride levels, serum insulin level, systolic blood pressure and diastolic blood pressure) were additionally included. Similar analyze were calculated for CAC × VE interaction. In the third model, the main effects of age, sex, VE, and CAC, as well as the interaction of CAC with VE, were included in the regression model testing the interaction between CAC and VE. In the forth model, the main effects of the risk factors were additionally included. The analyses were carried out in the whole sample and in the low, medium and high FMD (Table 2) and CAC groups (Table 3) separately.

When FMD was categorized, the lowest 30% and the highest 30% were classified into groups of low (FMD: −1.96–5.54%; n = 479) and high (FMD: 10.02–24.84%; n = 479) FMD responses, respectively. The values between the 30th and 70th percentiles were considered as representing a medium FMD response (FMD: 5.55–10.01%; n = 638). The same method was used when categorizing the CAC values (low CAC: 0.25–1.75%/10 mmHg, n = 479; medium CAC: 1.76–2.55%/10 mmHg, n = 638; high CAC: 2.56–5.31(%/10 mmHg, n = 479). The analyses were conducted separately for the low, medium, and high FMD and CAC groups using the regression models described above. Vital exhaustion and ultrasound measures included in the interactions were centralized to avoid multicollinearity [27].

## 3. Results

The mean values for the study parameters are presented in Tables 1–3.

Linear regression analyses showed that VE was not related to IMT when the analysis was performed for all participants (p = 0.895, N = 1,596 for the main effect of VE), nor in any FMD or CAC group (p ≥ 0.426 and p ≥ 0.554, respectively). FMD was negatively marginally significantly related to IMT in the whole sample (β = −0.045; p = 0.075, N = 1,596). However, FMD was not related to IMT in any FMD group (p ≥ 0.276). Likewise, CAC was negatively marginally significantly related to IMT in the whole sample (β = −0.046; p = 0.053; N = 1,596). When the analyses were performed separately in different CAC groups, CAC was shown to be negatively marginally significantly related to IMT only among medium CAC individuals (β = −0.074; p = 0.061; N = 638). In addition, VE was negatively significantly related to FMD in the whole population (β = −0.056; p = 0.025; N = 1,596). However, VE was not related to FMD in any FMD group (p ≥ 0.301). VE was not related to CAC when the analysis was performed in the whole sample (p = 0.890, N = 1,596), nor in any CAC group (p ≥ 0.541).

### 3.1. Flow-Mediated Dilatation x Vital Exhaustion Interaction in Predicting Intima-Media Thickness

Table 4 presents the results of linear regression analyses of interactions of FMD with VE for IMT performed a) for all participants together and b) separately for participants with low, medium, and high FMD responses. The interaction of FMD and VE for IMT was found to be non-significant in the whole sample (p = 0.992) and remained non-significant after adjusting for additional cardiovascular risk factors (p = 0.704).

The FMD × VE interaction was significant on IMT (p = 0.044) only in subjects with a low FMD status, whereas this interaction was marginally significant (p = 0.092) after adjusting for cardiovascular risk factors. Further linear regression analyses conducted separately for high VE (N = 228) and low VE (N = 251) participants (median split) from the low FMD response group showed that, among participants with high VE, lower FMD responses predicted higher IMT (β = −0.147; p = 0.027; N = 228). This association was non-significant in low VE subjects from this FMD group (β = 0.053; p = 0.404; N = 251).

### 3.2. Carotid Artery Compliance × Vital Exhaustion Interaction in Predicting Intima-Media Thickness

Table 5 shows the results of linear regression analyses of interactions of CAC with VE for IMT (a) for all participants together and (b) separately for participants with low, medium, and high CAC values. The interaction of CAC and VE for IMT was non-significant when the analysis was carried out in the whole sample (p = 0.390) and this interaction was also non-significant in a model adjusted with cardiovascular risk factors (p = 0.574). When the analyses were performed separately for low, medium, and high CAC individuals, the CAC × VE interaction was significant (p = 0.039) for IMT only in participants with a low CAC status. Further linear regression analyses performed separately for the high (N = 247) and low VE (N = 232) individuals (median split) from the low CAC group indicated that low CAC predicted high IMT levels only marginally significantly among high VE participants (β = −0.112; p = 0.067; N = 247), and was unrelated to IMT in low VE participants (β = 0.013; p = 0.849; N = 232). The interaction of CAC and VE for IMT was non-significant in a model adjusted with cardiovascular risk factors in the low and medium CAC responses group (p = 0.423 and p = 0.845, respectively) and marginally significant in the high CAC responses group (p = 0.077).

## 4. Discussion

According to the response-to-injury model of atherosclerosis, impaired systemic endothelial function indicating by a low FMD response [7] is an early dysfunctional factor for atherosclerosis development [2]. In line with this model, we found in the current study a significant interaction between FMD and VE for IMT only among participants with low FMD values, not among those who had medium or high FMD values that may indicate good vascular health. Our results imply that VE was related to an increased carotid IMT only among subjects with the very lowest FMD response.

These results are in line with the findings that long-term psychosocial stress, such as chronic life stress, accelerates atherosclerosis [3]. To our knowledge, the combined effects of VE and the standard atherosclerosis risk factors are not well-documented. Only a few studies have related VE components to ultrasonic measurements of carotid atherosclerosis. In the Kuopio Ischemic Heart Disease study, hopelessness (a component of VE) was related to increased carotid IMT among 900 middle-aged men [28]. Likewise, negative feelings (a part of VE) have been shown to be related to increased progression of carotid IMT in 94 men with hypertension [29]. In addition, chronic work stress has been associated with increased IMT levels in the CRYF study [13]. On the other hand, in the CRYF study, we have previously shown that brachial FMD is inversely related to carotid IMT [22]. In addition, we have observed that young adults with high levels of cardiovascular risk factors are at increased risk of having thickened carotid IMT’s, especially when they have evidence of endothelial dysfunction (impaired brachial FMD) [22].

Additionally, VE was found in the present study to be negatively related to FMD in the whole population. This suggests that VE may contribute to endothelial dysfunction. This suggestion is in agreement with the findings demonstrating that chronic psychosocial stress impairs endothelial function [14]. However, VE was not related to FMD in low FMD group. It seems that this association should be tested in a larger sample of low FMD individuals. Future studies are needed to explore possible causal effect of VE on vascular health.

The mechanisms of translations of chronic stress, including VE, into vascular damages and atherosclerosis progression are not fully established. Appels suggested that VE is causally related to the coronary heart disease events through the neuroendocrine mechanisms [15]. It has been found that VE leads to changes in sympathetic-parasympathetic balance [30] and in the tone of hypothalamic-pituitary-adrenal (HPA) axis, thereby decreasing stress coping [31]. These chronic stress-evoked alterations may influence the functioning of cardiovascular system and, therefore, accelerate the atherosclerotic process [32]. Both systems (HPA axis and sympathetic-parasympathetic balance) may contribute to vascular disease development and, possibly, may trigger endothelial dysfunction; and these two systems, as well as inflammatory mediators and also cross-talk interactions between these systems, are included into the pathogenesis of atherosclerotic process [32].

The second important finding of the present study was a significant interaction effect between CAC and VE on IMT in participants with low CAC values. Our results are in line with the previous findings that measurements of arterial compliance may offer evidence of vascular changes that occur in the first phases of atherosclerotic disease process [8] and that reduced arterial compliance reflects early functional atherosclerosis [33].

In line with our hypothesis, high VE was related to high IMT in subjects with low arterial elasticity (reflected by a low CAC level). This finding is in line with the concept that chronic stress contributes to the functioning of endothelium [14], however, the combined effect of chronic stress assessed by VE and arterial compliance on atherosclerosis has not previously been investigated. Reduced CAC reflects structural abnormalities of the vascular wall which are associated with aging and disease states [34]. The mechanism explaining the interactive effect of VE and arterial elasticity on atherosclerosis is not fully established. It is known that VE may elicit alterations in the sympathetic-parasympathetic balance [30] or in the tone of the HPA axis [31]. These systems may play a role in the development of endothelial dysfunction [32]. Endothelial dysfunction may, in turn, lead to structural alterations of the walls of vessels increasing the sensitivity of vasculature to the harmful influences of risk factors [34].

In the present study, controlling for the cardiovascular risk factors attenuated the results on FMD × VE interaction on IMT or CAC × VE interaction on IMT to non-significant which implies that the effect of FMD × VE interaction on IMT or CAC × VE interaction on IMT may be mediated through the risk factors. We may also suggest that FMD or CAC may be influenced by the risk factors. Thus, it has been reported in the previous CRYF studies, that brachial FMD response is related with HDL-cholesterol and with blood pressure [22,33]; and LDL-cholesterol and blood pressure are related with arterial elasticity [23,33]. Future studies are needed to shed light to complex causal effects.

No significant interactions of VE with FMD and CAC for IMT were found among participants with medium or high FMD or among participants with medium and high CAC, as well as in the whole population. However, this was not an unexpected finding. The relations between VE and heart diseases are very complicated. Healthy subjects may often feel themselves vitally exhausted; however exhaustion itself may have no additional impact to the increased disease risk. Different situation occurs among patients having heart disease or evidence for heart disease. First, a number of studies have demonstrated associations between VE and coronary events [15–18]. Second, it has been reported that undiagnosed (or preclinical) heart disease may lead to symptoms of exhaustion [35]. Based on this data, our finding on VE in combination with poor vascular health influencing the atherosclerotic processes but not among participants with good parameters of endothelium functioning (medium or high FMD and medium or high CAC) seems logical. The vascular health status has been suggested to modify the association between risk factors and atherosclerosis; possible, good vascular health even in a combination with high VE is a favorable factor in relation to the risk of atherosclerosis development. No significant interactions were found in the overall group. Possibly, this is resulted from high prevalence of participants with good vascular health parameters in the current data derived from a population based sample.

According to the present results, chronic stress assessed by VE may be one of the factors influencing the atherosclerotic processes in combination with poor vascular health. The significant interactions between FMD and VE, and between CAC and VE, for IMT were found only in participants with the very lowest FMD and CAC. We can conclude that VE may be harmful in young adults with evidence of endothelial dysfunction or reduced arterial elasticity and therefore may influence the atherosclerotic progression in early life.

## 5. Methodological Considerations

It is important to note some limitations of the present study. First, we measured carotid IMT from the left carotid artery, not from the internal carotid artery. Multipart determination of carotid IMT involving both internal and common carotid measurements might be more sensitive to atherosclerosis development [36]. However, it is known that the common carotid artery IMT is easily visualised and a highly reproducible measurement. The reproducibility of our measurements is comparable with other reports [37]. Previous data support the use of the common carotid artery IMT in both studies of risk factor associations and cohort studies [37,38].

Second, we found relatively large within-subject long-term variation in FMD [22] and CAC [23] measurements, but the variability in FMD and CAC responses is actually very comparable to that in some earlier reports [39–42]. The long-term reproducibility of the carotid and brachial diameter measurements was very good, which suggests that much of the variation in FMD and CAC is due to physiological fluctuation. It has been described in the literature, that several factors may affect FMD variation [43]. However, part of the variability naturally relates to the error of the method. The large variability of FMD and CAC is a limitation, and the results should be interpreted with caution until replicated in future research.

The present analysis was conducted in participants aged 24 to 39. Our results cannot be generalized to older individuals with more definite atherosclerosis. Also, owing to the cross-sectional nature of the present study, it is impossible to make statements regarding the atherosclerosis progress.

## 6. Conclusions

Vital exhaustion was shown to exert an effect on IMT in subjects with impaired endothelial responses. The findings are consistent with the idea that risk factors are more harmful if endothelium is not working properly.

## Figures and Tables

**Table 1.** Characteristics of study participants.

***Variable***	***Mean***	***(SD)***	***N***
Age in 2001, years (24–39)	31.59	5.05	1,596
Baseline brachial diameter (mm)	3.47	0.57	1,596
Flow-mediated dilatation, FMD (%)	8.04	4.44	1,596
Carotid artery compliance, CAC (%/10 mmHg)	2.20	0.74	1,596
LDL-cholesterol (mmol/L)	3.27	0.85	1,591
HDL-cholesterol (mmol/L)	1.31	0.32	1,591
Triclycerids (mmol/L)	1.30	0.83	1,592
Insulin (mU/L)	7.79	5.50	992
Systolic blood pressure (mm Hg)	117.08	13.12	989
Diastolic blood pressure (mm Hg)	70.98	10.86	989
Carotid intima-media thickness, IMT (mm)	0.58	0.09	1,596
Vital Exhaustion, VE	0.42	0.38	1,596

**Table 2.** Study characteristics in subgroups stratified by FMD response.

	***Low FMD***			***Medium FMD***			***High FMD***		

***Variable***	***Mean***	***(SD)***	***N***	***Mean***	***(SD)***	***N***	***Mean***	***(SD)***	***N***

Age, years (24–39)	31.22	5.26	479	31.57	5.07	638	31.98	4.75	479
Baseline brachial diameter (mm)	3.69	0.59	479	3.51	0.56	638	3.19	0.45	479
Flow-mediated dilatation,FMD (%)	3.14	1.72	479	7.75	1.26	638	13.34	2.89	479
LDL-cholesterol (mmol/L)	3.25	0.85	479	3.27	0.87	636	3.29	0.84	476
HDL-cholesterol (mmol/L)	1.29	0.30	479	1.29	0.32	636	1.35	0.33	476
Triclycerids (mmol/L)	1.25	0.68	479	1.36	0.86	637	1.29	0.90	476
Insulin (mU/L)	7.91	5.99	316	7.80	5.29	391	7.64	5.23	285
Systolic blood pressure (mm Hg)	117.55	13.46	317	115.80	12.54	386	118.29	13.40	286
Diastolic blood pressure (mm Hg)	71.92	11.06	317	69.93	10.26	386	71.36	11.32	286
Carotid intima-media thickness, IMT (mm)	0.58	0.09	479	0.58	0.10	638	0.57	0.09	479
Vital Exhaustion, VE	0.39	0.35	479	0.42	0.38	638	0.44	0.39	479

**Table 3.** Study characteristics in subgroups stratified by CAC response.

	***Low CAC***			***Medium CAC***			***High CAC***		

***Variable***	***Mean***	***(SD)***	***N***	***Mean***	***(SD)***	***N***	***Mean***	***(SD)***	***N***

Age, years (24–39)	33.26	4.78	479	31.45	5.05	638	30.10	4.81	479
Carotid artery compliance, CAC (%/10 mmHg)	1.39	0.28	479	2.15	0.23	638	3.09	0.45	479
LDL-cholesterol (mmol/L)	3.45	0.88	478	3.24	0.88	635	3.13	0.75	478
HDL-cholesterol (mmol/L)	1.26	0.33	478	1.31	0.31	635	1.35	0.31	478
Triclycerids (mmol/L)	1.49	0.94	479	1.28	0.89	635	1.14	0.52	478
Insulin (mU/L)	7.92	4.71	307	7.46	4.69	390	8.08	7.04	295
Systolic blood pressure (mm Hg)	117.64	13.52	304	116.40	12.78	389	117.40	13.16	296
Diastolic blood pressure (mm Hg)	70.49	10.88	304	70.71	10.77	389	71.85	10.93	296
Carotid intima-media thickness, IMT (mm)	0.59	0.10	479	0.58	0.09	638	0.58	0.10	479
Vital Exhaustion, VE	0.42	0.38	479	0.42	0.38	638	0.42	0.37	479

**Table 4.** Regression analysis of interaction between flow-mediated dilatation and vital exhaustion in predicting IMT.

	R^2^*	β**	R^2^*** change	**p**	**N**

**All FMD responses**					

FMD x VE^1^	0.129		0.000	0.992	1,596
#FMD x VE^1^	0.177		0.000	0.704	1,596

**Low FMD responses (30%)**					

*FMD x VE*	*0.139*		***0.007***	***0.044***	*479*
*sex*		*0.063*		*0.349*	
*age*		*0.318*		*0.000*	
*baseline brachial diameter*		*0.181*		*0.007*	
*FMD*		*−0.083*		*0.185*	
*VE*		*0.141*		*0.103*	
#FMD x VE^1^	*0.210*		*0.007*	*0.092*	*479*

**Medium FMD responses (40%)**					

FMD x VE^1^	0.131		0.000	0.992	638
#FMD x VE^1^	0.187		0.004	0.186	638

**High FMD responses (30%)**					

FMD x VE^1^	0.125		0.000	0.726	479
#FMD x VE^1^	0.199		0.002	0.408	479

VE-vital exhaustion

FMD-flow-mediated dilatation (%)

1 Main effects were included in each analysis but they are presented for low FMD group only

* Calculated for the whole model

** Refers to main effects

*** Refers to the interaction term

# Additionally adjusted for cardiovascular risk factors: LDL-cholesterol, HDL-cholesterol, triglycerides, insulin, systolic and diastolic blood pressure

**Table 5.** Regression analysis of interactions between carotid artery compliance and vital exhaustion in predicting IMT.

	R^2^*	β**	R^2^*** change	**p**	**N**
**All CAC responses**					
CAC × VE^1^	0.126		0.000	0.390	1596
#CAC × VE^1^	0.181		0.000	0.574	1596
**Low CAC responses (30%)**					
*CAC* × *VE*	*0.152*		***0.008***	***0.039***	*479*
*sex*		*0.146*		*0.001*	
*age*		*0.354*		*0.000*	
*CAC*		*−0.096*		*0.119*	
*VE*		*0.495*		*0.031*	
#CAC × VE^1^	0.212		0.000	0.423	479
**Medium CAC responses (40%)**					
CAC × VE^1^	0.107		0.000	0.799	638
#CAC × VE^1^	0.201		0.000	0.845	638
**High CAC responses (30%)**					
CAC × VE^1^	0.150		0.004	0.117	479
#CAC × VE^1^	0.194		0.009	0.077	479

VE-vital exhaustion

CAC-carotid artery compliance (%/10mmHg)

1 Main effects were included in each analysis but they are presented for low CAC group only

* Calculated for the whole model

** Refers to main effects

*** Refers to the interaction term

# Additionally adjusted for cardiovascular risk factors: LDL-cholesterol, HDL-cholesterol, triglycerides, insulin, systolic and diastolic blood pressure
